# The effect of a *Bacillus*-based probiotic on feed intake and digestibility of a forage and a feedlot diet in *Bos indicus* steers

**DOI:** 10.1016/j.vas.2025.100463

**Published:** 2025-05-19

**Authors:** Karen E. Eyre, Liyi Pan, Karen Harper, Luis F. Prada e Silva

**Affiliations:** The University of Queensland, Gatton, Queensland 4343, Australia

**Keywords:** Cattle, Direct-fed microbial, Fibre fermentation, Low-quality diet, Nutrient utilisation, Rumen

## Abstract

Ionophores are commonly used in feedlot rations to improve feed efficiency and reduce acidosis risk, particularly during the transition from forage to cereal-rich diets. With increasing concerns over non-therapeutic antibiotic use, interest in probiotics as potential alternative has grown. This study evaluated the effect of a *Bacillus*-based probiotic on feed intake and total tract nutrient digestibility in *Bos indicus* steers. Two diets were tested: a high-fibre forage diet (Rhodes grass hay, 690 g/kg neutral detergent fibre (NDF)) and a feedlot diet (20 % Rhodes grass hay, 40 % finisher pellet, 30 % peanut hull fibre pellet, 10 % ground barley, 470 g/kg NDF). Twelve steers (267 ± 7.5 kg) were blocked by body weight and randomly assigned to a control (*n* = 6) or a probiotic-supplemented group (*n* = 6) receiving 3 g per steer of *Bacillus licheniformis* 809 and *Bacillus subtilis* 810 (3.2 × 10^9^ CFU/g) for the trial duration. Each diet was fed for 34 days, followed by a 7-day sampling period for total faecal and urine collection. Probiotic supplementation increased organic matter digestibility by 7.8 % and NDF digestibility by 8.4 %, resulting in a significant increase (*P* < 0.05) in digestible fibre intake on the forage diet. No effects on intake or digestibility were observed on the feedlot diet. Probiotic supplementation did not affect nitrogen metabolism. While intake was unchanged, improved fibre digestibility suggests Bacillus-based probiotics can enhance nutrient utilisation in forage-based diets, potentially improving performance in high-fibre diets.

## Highlights

• Bacillus-based probiotic improved fibre digestibility in forage diets by over 8 %.
• No changes in intake or digestibility were observed with the feedlot diet.
• Probiotic supplementation did not affect nitrogen metabolism in Bos indicus steers.
• Enhanced nutrient utilisation in forage diets suggests performance benefits in cattle.

## 1 Introduction

Feed additives are often included in supplements to grazing cattle and in feedlot diets to improve animal health and productivity (Terry et al., 2020). Due to public health concerns regarding residues in animal products, probiotics have been identified as a replacement for antibiotics, including ionophores, in supplements and total mixed rations (Niranjan et al., 2023). Probiotics are live organisms that, when administered at an adequate level, provide a health benefit to the animal (FAO/WHO, 2006). In ruminants, the inclusion of probiotics in the diet has been shown to improve rumen fermentation, support immune function and improve production efficiency in beef and dairy cattle (Krehbiel et al., 2003). There has been a lot of focus in ruminants on *Lactobacillus spp, Saccharomyces cerevisiae* and *Bifidobacterium* but other species are also used (Mansilla et al., 2023; Parra et al., 2021).

Two of these are *Bacillus subtilis* and *B. licheniformis,* which are spore-forming, gram-positive bacteria which produce a range of fibrolytic, amylolytic and proteolytic enzymes (Elshaghabee et al., 2017; Su et al., 2020). These organisms are of growing interest not only as antibiotic alternatives but also for their potential to directly enhance nutrient digestibility through enzyme activity and microbial stimulation. Recent studies support this potential. Supplementation with *B. licheniformis* and *B. subtilis* improved feed efficiency in finishing bulls, despite a reduction in intake, and decreased plasma urea-N concentration, suggesting enhanced nitrogen utilisation (Cordeiro, Cappellozza, de Melo, & Bernardes, 2024).

In forage-based systems, *Bacillus*-based direct-fed microbials have been shown to increase intake, total-tract digestibility of dry matter, crude protein and fibre, and reduce plasma urea-N, indicating improved nutrient capture and possibly reduced nitrogen waste (Limede et al., 2025). Together, these results suggest that *Bacillus* supplementation may benefit both high- and low-energy diets, with responses likely influenced by forage quality, fermentable energy supply, availability and recycling of rumen-degradable nitrogen, and microbial ecology.

Pan et al. (2022) and Cappellozza et al. (2023) found that the addition of *Bacillus* spp. *in vitro* increased NDF digestibility in a range of tropical and temperate forages and starch digestibility in a variety of grains. The objective of this study was to test the impact of supplementing cattle with *Bacillus* strains on forage and a typical higher starch feedlot diet on intake and digestibility. We hypothesise that *Bacillus* supplementation will increase NDF digestibility in the forage diet and improve whole-diet digestibility in the feedlot diet.

## 2 Materials and methods

### 2.1 General

All experimental procedures involving animal care and management were approved by the University of Queensland Animal Ethics Committee (2021/AE000512). This experiment was conducted at the Queensland Animal Science Precinct, University of Queensland, Gatton, QLD from November 2022 until February 2023.

### 2.2 Animals and experimental diets

Twelve healthy, non-castrated Brahman (*Bos indicus*) steers (initial BW = 267 ± 7.5 kg), aged approximately 10–12 months, were sourced from a single cohort raised on Buffel grass (*Cenchrus ciliaris* L.) pasture with mineral supplementation. Upon arrival, steers were weighed and treated with a pour-on anthelmintic (CattleGuard, Zoetis Australia Pty Ltd, Rhodes, NSW) as part of the induction process. They were then fed a forage-based diet until the start of the trial.

Steers were blocked by initial BW and placed in individual pens with overhead shade and automatic waterers. Blocking was used to account for residual variation in metabolic demands associated with initial BW, even though the group was relatively homogeneous. The modelled difference in initial BW between blocks was approximately 13 kg, and while not statistically significant, this variation justified retaining block as a random effect in the model.

The experiment consisted of two consecutive periods. During the first period (d 1 to 42), steers were fed a forage diet. During the second period (d 43 to 99), steers were transitioned to and then maintained on a high-starch feedlot diet. Following the metabolism phase, steers were transitioned to the feedlot diet using a two-step adaptation protocol. From d 43 to 51, steers received a transitional diet containing 34 g/kg starch and 623 g/kg NDF (Adaptation 1), followed by a second adaptation diet from d 52 to 58 containing 120 g/kg starch and 553 g/kg NDF (Adaptation 2). On day 59, steers began receiving the final feedlot diet, which contained 200 g/kg starch and 470 g/kg NDF. The dietary composition is shown in Table 1.

**Table 1:** Ingredients and chemical composition of forage and high starch diets.

Item	Forage Diet	Adaptation 1	Adaptation 2	Feedlot Diet
Ingredient, g/kg DM				
Rhodes grass hay	980	700	300	200
Barley grain (ground)	15		100	100
Finisher Pellet^1^		100	200	400
High Fibre Pellet^2^		195	395	295
Mineral premix^3^	5	5	5	5
Analysed nutrient composition				
DM (g/kg)	851	863	878	879
OM, g/kg DM	908	919	939	918
CP, g/kg DM	125	121	129	132
NDF, g/kg DM	684	623	553	483
Ether extract, g/kg DM	16	19	23	24
Starch, g/kg DM	12	34	121	160

1 Finisher pellet composition: 890 g/kg DM, 114 g/kg CP, 230 g/kg NDF, 300 g/kg Starch, 27 g/kg Fat, 12.4 MJ ME/kg DM, 570 g/kg barley, 100 g/kg wheat, 200 g/kg chickpea shell, 50 g/kg millrun, 25 g/kg bentonite, 20 g/kg molasses, 15 g/kg Aglime, 8 g/kg sodium bicarbonate, 6.25 g/kg urea, 5 g/kg vegetable oil, 3 g/kg salt, 1.5 g/kg ammonium sulphate, 1 g/kg mineral premix.

2 High Fibre Pellet composition: The main ingredient is peanut shell, with 880 g/kg DM, 87 g/kg CP, 520 g/kg NDF, 40 g/kg Fat, 9.4 MJ ME/kg DM.

3 Mineral premix: 280 g/kg dicalcium phosphate, 200 g/kg bentonite, 150 g/kg salt, 114.6 g/kg canola meal, 75 g/kg soya meal, 51.5 g/kg lime, 40 g/kg gypsum, 15 g/kg magnesium oxide, 10 g/kg molasses, 2 g/kg mineral premix, 1.5 g/kg vitamin premix, 0.4 g/kg flavophospholipol.

Steers remained on the final feedlot diet for 41 days (d 59 to 99). Intake and body weight data were collected from d 59 to 92, followed by a 7-day metabolism trial in individual crates from d 93 to 99. The same treatment allocations (Control or Bovacillus) were maintained throughout the adaptation period and Phase 2.

The two dietary treatments were 1) Control, no probiotic (*n* = 6) and 2) Treatment (*n* = 6), 3 g/d of a probiotic (Bovacillus; Chr. Hansen A/S, Hørsholm, Denmark) containing a mixture of *Bacillus licheniformis* 809 (1.6 × 10^9^ CFU/g) and *Bacillus subtilis* 810 (1.6 × 10^9^ CFU/g); mixed with 100 g of ground barley. Control animals also received 100 g of ground barley daily to ensure equal supplement volume across groups. The calculated dose of 3 g/d has been shown to increase fibre digestibility in an *in vitro* system (Pan et al., 2022) and is recommended by the manufacturer.

The treatments were randomly assigned within each block, and steers remained on their assigned treatment for the full trial. The study was designed as a randomised block design, with six steers per treatment group. Based on a coefficient of variation of 3.5 % for daily intake and 2.5 % for apparent total tract NDF digestibility (Parra et al., 2021), the minimum detectable difference was estimated at 6 % for dry matter intake and 4.5 % for NDF digestibility (α = 0.05, β = 0.80).

Control and treatment steers were separated by an empty pen to prevent cross-contamination. All animals received a commercial mineral and vitamin mix that did not contain ionophores. Steers were fed each diet *ad libitum* from d 1 to 35 and d 43 to 78, with residuals collected and weighed, and fresh feed provided at approximately 8:00 am daily. The amount fed was adjusted daily to achieve a feed residual of approximately 50 g/kg of the offered amount. Steers were weighed every 14 d on two consecutive days before feeding to minimize rumen fill, but feed was not withheld before weighing.

### 2.3 Digestibility measurement

On d 36 and 79, all 12 steers were moved into metabolism crates for 7 d Each crate was raised and designed with a gentle slope towards the rear, featuring a split floor design. The front was covered with a rubber mat for steer comfort, while the rear end had a slatted floor that allowed faeces and urine to pass through (Silva et al., 2021). Steers were fed the same diet at 90 % of their *ad libitum* intake. Nutrient digestibility was measured over the final 5 d A daily sample of the diet fed was collected and combined into a composite sample representing the 5 d-period. Daily residuals were collected, weighed and combined into a composite sample for each steer.

Total faecal output was collected, thoroughly mixed, and weighed daily. A 10 % subsample was collected each day and refrigerated; these daily subsamples were then composited for each steer at the end of the 5-day collection period. Urine was collected in bins located beneath each crate, fitted with a 2 mm screen to prevent faecal contamination, and pumped into containers containing 50 mL/L H₂SO₄ to reduce pH to <4 and inhibit bacterial activity. Urine volume was measured daily, and a 10 % aliquot was taken, composited across days, and refrigerated until the end of the collection period. At the end of the period, representative subsamples of diet, orts, faeces, and urine were thoroughly mixed and retained for analysis.

### 2.4 Analysis

All samples of hay and supplements, refusals, and faeces from both experiments were dried at 60 °C for 72 h in a forced-air oven for analysis and then at 105 °C to determine dry matter (DM). All samples were ground through a 2 mm screen (Retsch ZM 200; Haan, NW, Germany). All samples were incinerated at 550 °C for 8 h (Modutemp, Perth, WA, Australia) and the ash content used to determine organic matter (OM) Ash-free neutral detergent fibre (aNDF) was determined using alpha-amylase by an adaptation of the procedure of Van Soest et al. (1991) on an Ankom 200 fibre analyser (Ankom Technology Corporation, Fairport, NY, USA). The crude fat content was measured by the ether extract content after extraction with petroleum spirit on a SER148 Solvent Extraction Unit (VELP Scientifica S.R.L, Usmate, MI, Italy). The nitrogen concentration of diet, urine and faeces was determined using the Dumas combustion method (Sweeney, 1989) on a LECO CN928 combustion analyser (LECO Corporation, St Joseph, MI, USA). The N concentration was multiplied by 6.25 to get crude protein (CP).

Supplements, refusals, and faeces from Phase 2 (Feedlot Diet) were analysed for starch content. Starch was analysed using an enzymatic colorimetric assay following the method of Karkalas (1985). Samples and standards were incubated for 45 min and read with a standard 96-well colourimetry plate reader.

### 2.5 Calculations and statistical analysis

Apparent total-tract digestibility of DM, OM, NDF, and starch was calculated from the relevant nutrient intake and faecal flow. N retention was calculated as total N intake minus N excretion in faeces and urine. Nitrogen use efficiency (NUE) was calculated as the proportion of retained N (g/day) divided by digested N (g/day) (Archibeque et al., 2001).

Initial and final BW, as well as intake data collected during the 34 days preceding the metabolism trial, were analysed using linear mixed models in the MIXED procedure of SAS (version 9.4). The models included treatment as a fixed effect and block as a random effect, with individual steer as the experimental unit. For intake data, day was included as a repeated measure using an AR(1) covariance structure, selected based on the lowest Bayesian Information Criterion. Degrees of freedom were adjusted using the Kenward–Roger method.

Although the block effect was not statistically significant in most models (*P* > 0.30), it was retained when it contributed non-zero variance, as it helped account for residual variation related to animal allocation. In cases where the variance for block was estimated as zero, PROC MIXED profiled it out of the likelihood function and excluded it from the final model.

Because the two diets were administered sequentially (forage diet followed by feedlot diet), each phase was treated as an independent period. Consequently, no statistical comparison between diets was performed, and treatment effects (Control *vs.* Probiotic) were analysed separately within each period.

Total tract digestibility and nitrogen metabolism data were analysed as a randomised block design including the fixed effect of treatment (with or without *Bacillus* spp.) and the random effect of block, with individual steer as the experimental unit. Treatment effects were compared using Fisher’s least significant difference (option DIFF from LSMEANS), and significance declared at *P* ≤ 0.05 and trends at *P* ≤ 0.10.

## 3 Results

There was no significant effect (*P* > 0.10) of *Bacillus* probiotic supplementation on either initial or final BW or *ad libitum* DMI during the 34-d trial in either the forage or feedlot diets (Table 2). Steers maintained similar feed consumption and growth rates regardless of treatment.

**Table 2:** Effect of specific *Bacillus* strains supplementation on *ad libitum* dry matter intake and growth of steers receiving forage and high starch diets.

Item	Forage Diet				Feedlot Diet			
	Control	Probiotic	SEM	*P*-value^1^	Control	Probiotic	SEM	*P*-value^1^
DMI^2^ (kg/d)	5.7	5.6	0.13	0.37	8.6	9	0.2	0.12
Initial BW^3^ (kg)	266	275	13	0.55	308	306	12.6	0.87
Final BW^3^ (kg)	291	295	14	0.79	340	335	13.8	0.76

1 Treatment effects were analysed separately within each diet period. Block was included as a random effect.

2 DMI: dry matter intake.

3 BW: body weight.

When steers were fed the forage diet, *Bacillus* supplementation significantly increased (*P* < 0.01) both OM and aNDF digestibility. This improvement resulted in a 6 % increase (*P* < 0.01) in the daily amount of aNDF digested compared to control steers (Table 3). However, *Bacillus* supplementation did not affect (*P* > 0.10) any measure of nutrient intake or digestibility when steers were fed the high-starch feedlot diet (Table 3). Total tract starch digestibility approached 100 % in both groups, with no significant difference between control (99.3 %) and supplemented (99.8 ± 0.26 %) steers.

**Table 3:** Effect of specific Bacillus strains supplementation on intake and digestibility of steers receiving forage and high starch diets.

Item	Forage Diet				Feedlot Diet			
	Control	Probiotic	SEM	P-value^1^	Control	Probiotic	SEM	P-value^1^
Intake (kg/d)								
Dry matter	5.18	5.09	0.09	0.49	6.79	7.10	0.30	0.50
Organic matter	4.73	4.67	0.08	0.60	6.23	6.51	0.29	0.51
aNDF^1^	3.25	3.20	0.05	0.58	3.00	3.14	0.14	0.51
Crude protein	0.64	0.63	0.01	0.76	0.90	0.90	0.04	0.98
Starch	–	–	–	–	1.09	1.14	0.05	0.49
Digestibility (g/kg)								
Dry matter	511	535	6.3	0.01	582	583	9.0	0.92
Organic matter	499^a^	538^b^	8.9	<0.01	586	595	8.9	0.47
aNDF^2^	497^a^	539^b^	7.3	<0.01	349	368	13.1	0.32
Crude protein	467	463	5.8	0.74	500	493	17.4	0.74
Starch	–	–	–	–	993	998	2.6	0.18
Digestible aNDF intake^3^ (g/d)	1.61^b^	1.72^a^	0.02	<0.01	1.05	1.15	0.07	0.19

1 Treatment effects were analysed separately within each diet period. Block was included as a random effect.

2 Ash-free neutral detergent fibre.

3 Digestible aNDF intake was calculated as aNDF intake × aNDF digestibility.

a,b Values with different superscripts are significantly different *P* < 0.01.

Additionally, there was no significant difference (*P* > 0.10) in nitrogen intake, nitrogen excretion in either urine or faeces or NUE in steers supplemented with *Bacillus* strains in either diet (Table 4).

**Table 4:** Effect of specific Bacillus strains supplementation on Nitrogen intake and excretion of steers receiving forage and high starch diets.

Item	Forage Diet				Feedlot Diet			
	Control	Probiotic	SEM	*P*-value^1^	Control	Probiotic	SEM	*P*-value^1^
N intake (g/d)	102.9	101.7	1.66	0.60	142.8	147.0	7.65	0.71
N digestibility (g/kg)	534	539	7.4	0.67	506	516	22.8	0.72
Digested N (g/d)	55.1	54.6	0.89	0.62	71.6	76.1	5.77	0.59
Faeces N (g/d)	47.8	46.7	1.15	0.50	70.5	70.9	3.01	0.92
Urine N (g/d)	50.5	49.0	2.19	0.63	62.0	61.1	3.97	0.88
Total N excretion (g/d)	98.2	95.6	2.80	0.52	132.9	132.2	6.03	0.93
Faeces N (g/kg of N intake)	466	462	6.5	0.73	495	484	20.9	0.69
Urine N (g/kg of N intake)	491	483	20.8	0.79	430	421	20.8	0.77
Retained N (g/d)	4.71	5.91	2.35	0.72	10.1	15.3	4.77	0.46
Nitrogen use efficiency^2^	8.0	10.6	4.10	0.66	13.1	16.0	5.79	0.73

1 Treatment effects were analysed separately within each diet period. Block was included as a random effect.

2 Nitrogen use efficiency calculated as retained N over digested N.

## 4 Discussion

In support of our initial hypothesis, which proposed that *Bacillus* strains would enhance digestibility in forage diets, we observed significant increases in both OM and aNDF digestibility. Previous studies have primarily focused on the impact of lactate-utilising bacteria and the yeast *Saccharomyces cerevisiae* as probiotics for improving intake and performance in beef and dairy cattle (Adeyemi et al., 2019; Beauchemin et al., 2003; Chaucheyras-Durand & Durand, 2010; Guedes et al., 2008). Research on *Bacillus*-based probiotics in ruminants is limited, but there is evidence supporting increased nutrient digestibility, both *in vitro* (Cappellozza et al., 2023; Pan et al., 2022) and *in vivo* (Lopez et al., 2024).

The observed higher DM digestibility compared to OM digestibility in this study, for both the forage and feedlot diets, may be attributed to differences in the digestibility of minerals. The lower OM digestibility can be explained by the relatively higher digestibility of minerals in the diet, including those from the mineral supplement added at 5 kg per ton. In low-quality diets, such as the forage diet in the current study, minerals such as calcium and phosphorus can be digested at higher rates than organic matter. For instance, CSIRO (2007) reports true absorption coefficients for calcium and phosphorus of 0.68 and 0.70, respectively, which are greater than the average DM digestibility of the diet in the present study. This could lead to higher mineral absorption than total DM, explaining the higher DM digestibility values relative to OM.

The effects of *Bacillus* on aNDF digestibility are variable. *In vitro,* Pan et al. (2022) found that there was a mean increase in the NDF digestibility of a variety of Australian forages by 27.6 %, and Cappellozza et al. (2023) reported a 12.8 and 15.1 % improvement in NDF digestibility of individual forages at 24 and 48 h, respectively. Notably, the *in vitro* improvements in NDF digestibility were observed only when the NDF content of the substrate was greater than 32 % (Cappellozza et al., 2023; Pan et al., 2022). *In vivo* studies show variability in the response to *Bacillus* supplementation. In this study, an 8.4 % increase in NDF digestibility was observed in steers fed the forage diet (684 g NDF/kg DM), while no effect was detected in steers fed the feedlot diet (483 g NDF/kg DM). Similarly, Lopez et al. (2024) found a modest 5.3 % improvement in NDF digestibility with *Bacillus* supplementation in feedlot diets with low NDF content (maximum 280 g/kg). These findings suggest that Bacillus supplementation may have a greater impact on NDF digestibility in higher-fibre diets.

While some studies, including the current study, have demonstrated increases in NDF digestibility with *Bacillus* supplementation, others report no improvement, even in diets with high NDF content (Calaca et al., 2022; Dias et al., 2022). This variability in response suggests that the mechanism behind *Bacillus’s* influence on fibre digestibility may not solely depend on the NDF content of the diet. Instead, it could involve the synergistic activity of *Bacillus* strains, which enhance the cellulolytic capacity of the rumen microbiome (Wang et al., 2016). *Bacillus licheniformis* synthesises cellulases, while *B. subtilis* releases expansin-like proteins that disrupt hemi-cellulose-cellulose bonds, making cell-wall polysaccharides more accessible to cellulases (Liu et al., 2015; Pech-Cervantes et al., 2019).

Another potential mechanism for improved NDF digestibility is changes in ruminal fermentation, particularly through the increased production of branched-chain fatty acids (BCFA), which serve as substrates for cellulolytic bacteria (Roman-Garcia et al., 2021). Increased BCFA concentrations when using *Bacillus*-based probiotics have been reported by others (Calaca et al., 2022; Lamontagne et al., 2023; Sun et al., 2013; Wang et al., 2016). Lamontagne et al. (2023) also found that *Bacillus* supplementation altered the relative abundance of rumen microbial species, particularly those involved in soluble carbohydrate degradation.

Despite the improvement in aNDF digestibility, DMI did not increase in this study. Oba and Allen (1999) proposed that a faster rate of NDF digestion should lead to higher DMI and performance. However, the lack of an increase in NDF intake, despite improved digestibility with *Bacillus* supplementation, may be explained by two possibilities. First, the enhanced fibre digestibility may have accelerated fibre breakdown and clearance in the rumen, but this alone may not have been sufficient to stimulate additional intake, with total rumen fill still constraining intake. A study by Pereira et al. (2021) provides evidence for this concept. They evaluated the effects of silage source and undigested NDF concentration in beef finishing diets and found that even small increases in undigested NDF altered rumen fill, despite the lack of effect in daily intake. Second, the energy yield from enhanced fibre digestibility may have contributed to metabolic feedback signals that reduce the drive for further intake, particularly through hepatic oxidation of substrates like propionate and butyrate, cancelling the stimulus for greater intake (Allen et al., 2009).

There was no improvement in growth performance on either diet despite the increase in NDF digestibility in the forage-only portion of the trial. The intake and growth portion of the trial was only 35 d as the trial was primarily designed to test the effect on intake and digestibility rather than performance. Dias et al. (2022) found that *Bacillus* supplements tended to increase both average daily gain and significantly improved feed conversion efficiency in *Bos indicus* bulls on a high-energy feedlot diet, while Lopez et al. (2024) found a tendency for increased carcass weight with a combination of probiotics but no increase in BW.

Individual feedstuffs were assessed for starch digestibility by Pan et al. (2022), who reported that starch digestibility increased by 6.0 to 18.2 % when incubated with *B. licheniformis* and *B. subtilis.* No change in starch digestibility was observed in the current study, which agrees with the *in vitro* assessment of total mixed rations by Cappellozza et al. (2023). There was almost complete digestion of starch by both the control and treatment steers in this experiment (99.3 and 99.8 %, respectively), so there was no capacity for increased starch digestion. However, there may have been effects on rumen fermentation parameters and post-ruminal metabolism that were not measured in this experiment.

Although total nitrogen intake did not differ between treatments, the increase in OM digestibility could have enhanced the fermentable energy supply to rumen microbes. This would typically increase microbial protein synthesis and nitrogen utilisation efficiency (Silva et al., 2019). However, no changes were observed in nitrogen retention or NUE, suggesting either that nitrogen supply was not limiting microbial growth, or that any changes in microbial utilisation were insufficient to alter whole-animal N balance under the conditions of this study.

No effects on nutrient intake or digestibility were observed in beef steers on a feedlot diet supplemented with *Bacillus*, but there was a lower acetate:propionate ratio and rumen ammonia concentration, with a numerical trend towards higher pH levels post-feeding (Dias et al., 2022). These findings, along with the lack of response in digestibility in the feedlot diet, indicate that the second part of our hypothesis, improved whole-diet digestibility under high-starch conditions, was not supported. As described in the Methods, treatment effects were analysed separately for each diet period due to the sequential nature of the trial, and no direct comparison between forage and feedlot diets was performed.

Overall, the improvement in fibre digestibility in the forage diet highlights the potential role of Bacillus-based probiotics in enhancing nutrient utilisation in high-fibre diets, though further research is needed to explore their effects on rumen fermentation, post-ruminal digestion, and overall cattle performance. The lack of treatment effects on intake, nitrogen metabolism, and performance may reflect the relatively small sample size, short duration of the trial, and limited ability to measure microbial or post-ruminal responses.

## 5 Conclusion

This study tested the hypothesis that Bacillus supplementation would increase NDF digestibility in a forage diet and improve whole-diet digestibility in a high-starch feedlot diet; however, only the first part of this hypothesis was supported. Supplementing steers with *Bacillus licheniformis* 809 and *Bacillus subtilis* 810 improved OM and aNDF digestibility by 7.8 % and 8.4 %, respectively, when steers were fed a forage diet, supporting the first part of our hypothesis. However, there was no effect of *Bacillus* supplementation on nutrient digestibility in the high-starch feedlot diet, indicating that the second part of our hypothesis was not supported. While we did not measure post-ruminal parameters or feed efficiency, the increased fibre digestibility on the forage diet suggests potential benefits in cattle systems where high-fibre diets are prevalent.

## Author statement

The authors declare that the studies were approved by The University of Queensland Animal Ethics Committee (2021/AE000512) and conducted in accordance with the Australian code for the care and use of animals for scientific purposes.

## Ethical statement

This study protocol was reviewed and approved by the Production and Companion Animals Animal Ethics Committee of the University of Queensland with protocol number 2021/AE000512 of 13/08/2021.

## CRediT authorship contribution statement

**Karen E. Eyre:** Writing – review & editing, Writing – original draft, Supervision, Project administration, Methodology. **Liyi Pan:** Writing – original draft, Methodology, Investigation. **Karen Harper:** Writing – review & editing, Supervision, Methodology. **Luis F. Prada e Silva:** Writing – review & editing, Supervision, Project administration, Methodology, Funding acquisition, Formal analysis, Conceptualization.

## Declaration of competing interest

The authors declare the following financial interests/personal relationships which may be considered as potential competing interests:

Luis Prada e Silva reports financial support was provided by Novonesis. If there are other authors, they declare that they have no known competing financial interests or personal relationships that could have appeared to influence the work reported in this paper.
